# Soy Phospholipids Exert a Renoprotective Effect by Inhibiting the Nuclear Factor Kappa B Pathway in Macrophages

**DOI:** 10.3390/metabo12040330

**Published:** 2022-04-06

**Authors:** Satoshi Ohta, Masashi Asanoma, Nao Irie, Nobuhiko Tachibana, Mitsutaka Kohno

**Affiliations:** 1 Research Institute for Creating the Future, Fuji Oil Holdings Inc., 4-3 Kinunodai, Tsukubamirai-shi 300-2497, Ibaraki, Japan; irie.nao@so.fujioil.co.jp (N.I.); tachibana.nobuhiko@so.fujioil.co.jp (N.T.); 2 Soy Ingredients R&D Department, Fuji Oil Co., Ltd., 1 Sumiyoshicho, Izumisano-shi 598-8540, Osaka, Japan; asanoma.masashi@so.fujioil.co.jp; 3 R&D Division Strategy Planning Department, Fuji Oil Co., Ltd., 1 Sumiyoshicho, Izumisano-shi 598-8540, Osaka, Japan; kohno.mitsutaka@so.fujioil.co.jp

**Keywords:** soy, phospholipids, genistein, kidney, inflammation, macrophage

## Abstract

Complications associated with chronic kidney disease (CKD), which involves kidney inflammation, are a major health problem. Soy protein isolate (SPI) reportedly inhibits CKD exacerbation; however, its detailed action mechanism remains obscure. Therefore, the role of the polar lipid component of SPI in suppressing inflammation was investigated. Zucker fatty rats were divided into three groups and fed a diet containing casein, SPI, or casein + SPI ethanol extract (SPIEE) for 16 weeks. The isoflavones and phospholipids of SPIEE were evaluated for their anti-inflammatory effects. Rats in the SPI and casein + SPIEE groups showed reduced levels of the urinary *N*-acetyl-β-d-glucosaminidase and renal IL-1β mRNA (an inflammatory marker) compared with those in the casein group. In proximal tubular cells, genistein significantly inhibited monocyte chemoattractant protein-1 (MCP-1) expression induced by an IL-1β stimulus. In macrophages, soybean phospholipids suppressed lipopolysaccharide-induced IL-1β gene expression by inhibiting the phosphorylation of inhibitor κB and p65. Phosphatidylinositol (PI) was found to be essential for inhibition of IL-1β expression. SPIEE inhibited the exacerbation of kidney disease. Genistein and soybean phospholipids, especially soybean-specific phospholipids containing PI, effectively inhibited the inflammatory spiral in vitro. Hence, daily soybean intake may be effective for inhibiting chronic inflammation and slowing kidney disease progression.

## 1. Introduction

The increasing incidence of chronic kidney disease (CKD) is a global concern. CKD lowers quality of life because of dietary restrictions and the need for dialysis treatments in patients with end-stage disease. Moreover, CKD influences the pathogenesis of cardiovascular diseases, increasing the risk of cardiovascular events and death [1]. The metabolic syndrome has also recently been recognized as a strong risk factor for CKD [2,3]. Therefore, first-line therapy for CKD remains to improve healthy habits, especially dietary management and daily exercise, to prevent disease progression.

Several epidemiological studies have demonstrated that the consumption of dietary fiber, vitamins, and polyunsaturated fatty acids is important to prevent CKD development [4,5,6]. Protein consumption is considered to impose a burden on kidney function; thus, protein intake is restricted as CKD pathology progresses. Indeed, delaying CKD progression has been achieved by reducing red meat (and animal protein) intake. Since proteins are vital nutrients for survival, the significance of protein quality in CKD should be considered [7].

Soy protein isolate (SPI), a typical source of plant protein, performs various physiological functions in lifestyle-related diseases, such as improving insulin resistance, decreasing serum triglyceride levels, and preventing the development of obesity. Specifically, the SPI component β-conglycinin (a vicilin storage protein of soybeans) is reportedly responsible for these functions [8,9,10]. Furthermore, consuming SPI instead of animal protein has been shown to inhibit CKD progression in vivo and in clinical human studies [11,12]. For instance, in a 4-year study conducted by Azadbakht et al. [13], when 50% of animal dietary protein was replaced with SPI in patients with type 2 diabetes, the urinary protein levels and inflammation decreased, while lipid metabolism improved. Moreover, Jheng et al. [14] reported that soy protein exerts anti-inflammatory effects on renal tubules and mitigates fibrosis in glomeruli. Chronic inflammation is assumed to be responsible for the aggravation of kidney disease, whereas anti-inflammatory action is considered remedial in preventing such aggravation [15,16]. A previous study showed that SPI had a protective effect on renal tubular disorders, such as hyaline cast formation disorder, in Zucker rats [17]. Thus, a hypothesis emerged that soybean inhibits kidney disease progression by inhibiting inflammation in renal tubules; however, its exact function has not yet been clarified.

SPI contains trace amounts of other components, such as isoflavones, saponins, and phospholipids (PLs), in addition to proteins that help decrease CKD progression. For example, genistein, an isoflavone component of SPI, prevents CKD development by inhibiting inflammation and fibrosis [18,19]. Alcohol-extracted low-isoflavone soy protein has also been reported to exert similar inhibitory effects on CKD; however, its detailed mechanism of action has not been clarified [20].

Previous studies on nephropathy have frequently used Zucker fatty rats. Sato et al. reported that the value of NAG in 17-week-old lean Zucker rats was <0.2 units (U)/day, whereas it was 0.5 U/day for Zucker fatty rats [21]. They also observed that the weights of the lean Zucker rats and Zucker fatty rats were <400 g and approximately 600 g, respectively; in contrast Trujillo et al. reported that the weights of lean and Zucker fatty rats were 440 g and 708 g, respectively [21,22]. These observations suggest that NAG values and excess weight are potential markers to determine whether the Zucker rat model of nephropathy is properly constructed. Hence, in Zucker fatty rats, these biomarkers may be used to evaluate the effect of different dietary components on CKD. 

This study aimed to elucidate the contribution of the insoluble SPI components in preventing CKD development and the mechanism by which they prevent inflammation in nephropathy. The CKD-suppressing effect of SPI ethanol extract (SPIEE) was investigated in vivo (using a Zucker rat model of obesity-related nephropathy). The active components of SPIEE were evaluated in vitro (using the immortalized rat tubular epithelial cell line NRK-52E and the human monocyte cell line THP-1).

## 2. Results

### 2.1. Composition of Isoflavones and Phospholipids in the Insoluble Fraction

Extraction and rinsing with hydrous ethanol were performed to extract phospholipids and isoflavones from SPI. As shown in Table 1, isoflavones and phospholipids were detected from SPIEE.

### 2.2. SPI and SPIEE Suppress Nephropathy in Zucker Rats

No significant differences were observed in the final body weight and the amount of food intake amount among the three groups after 16 weeks of the experimental diet (Table 2). The levels of alanine aminotransferase, a marker of hepatic disorder, were slightly (albeit non-significantly) elevated in the casein group but suppressed in the SPI and SPIEE groups (data not shown); therefore, liver inflammation was not induced in the animal model used in this study.

**Figure 1:**
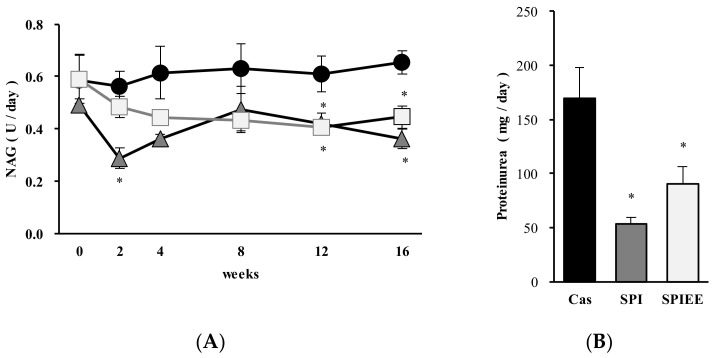
Nephroprotective effect of SPIEE in Zucker fatty rats. (**A**) Effects of the experimental diets on urinary NAG levels. Circles, triangles, and squares represent the casein, SPI, and SPIEE groups, respectively. (**B**) Urinary protein excretion after consumption of the experimental diets. * *p* < 0.05 vs. casein group.

Figure 1A shows the levels of NAG, which is a biomarker of renal tubular damage. The NAG levels in the SPI and SPIEE diet groups were lower than those in the casein group throughout the experimental period, and significant differences were noted in the 12th and 16th week. The levels of extracted urinary proteins in the SPI- and SPIEE-fed rats were significantly lower than those in casein-fed rats at the end of the trial (Figure 1B).

**Figure 2:**
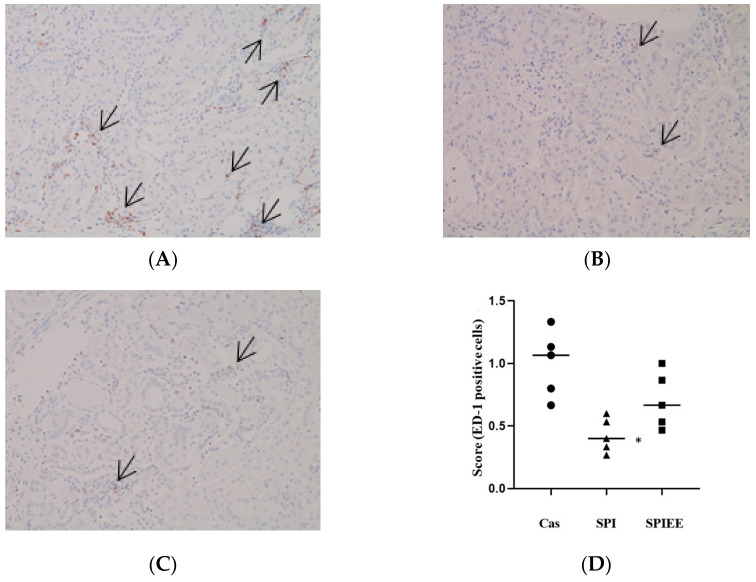
Pathological section of a kidney. To analyze the dietary effects on macrophage infiltration, kidney tissue sections were stained for ED-1. Arrows indicate ED-1-stained cells (**A**–**C**). (**D**) ED-1-positive cell scores were evaluated in 15 microscopic fields for each rat and are plotted for five rats. Circles, triangles, and squares represent the casein, SPI, and SPIEE groups, respectively. Bars indicate mean. * *p* < 0.05 vs. casein group.

Figure 2A–C shows the extent of macrophage infiltration as determined by immunostaining for ED-1. The mean positive cell scores in the casein, SPI, and SPIEE groups were 1.0, 0.4, and 0.7, respectively. The mean score in the SPI group was significantly lower than that in the casein group, whereas no significant difference was found between the SPIEE and casein groups (Figure 2D).

**Figure 3:**
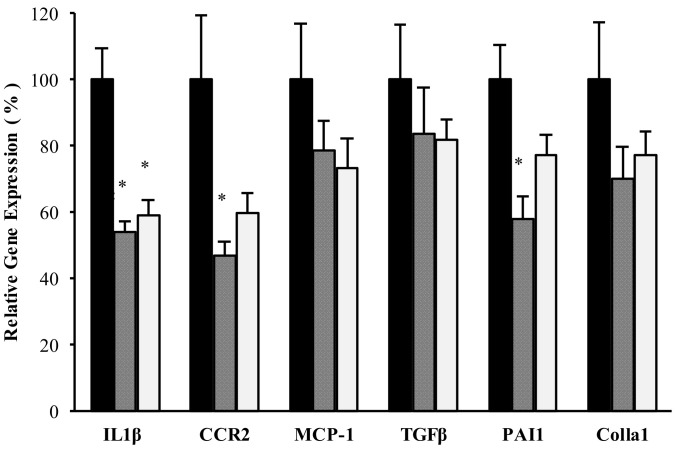
Renal mRNA levels after 16 weeks of experimental diet consumption. Target gene expression is shown relative to the expression level in the casein group. Black, dark gray, and light gray bars represent the casein, SPI, and SPIEE groups, respectively. Data are means ± SEs of five rats. * *p* < 0.05 vs. casein group.

Inflammation- and fibrosis-related gene expression in the kidneys was assessed at the end of the experimental period. MCP-1 and Col1a1 mRNA levels were markedly decreased in the SPI and SPIEE groups compared with those in the casein group; however, this difference was not statistically significant (Figure 3). CCR2 and PAI-1 mRNA levels were significantly lower in SPI-fed rats than in casein-fed rats. IL-1β mRNA levels were significantly lower in the SPI and SPIEE groups than in the casein group (Figure 3). TGF-β mRNA levels were lower in the SPI and SPIEE groups than in the casein group; however, this difference was not statistically significant (Figure 3).

Together, these data showed that SPIEE consumption significantly suppressed NAG, proteinuria, and IL-1β mRNA levels to a similar extent as SPI consumption. Further, SPI consumption significantly suppressed macrophage infiltration and inflammatory gene expression (CCR2 and MCP-1), with similar effects observed for SPIEE consumption.

### 2.3. PLs Suppress IL-1β Expression in Macrophages via the NF-κB Pathway

**Figure 4:**
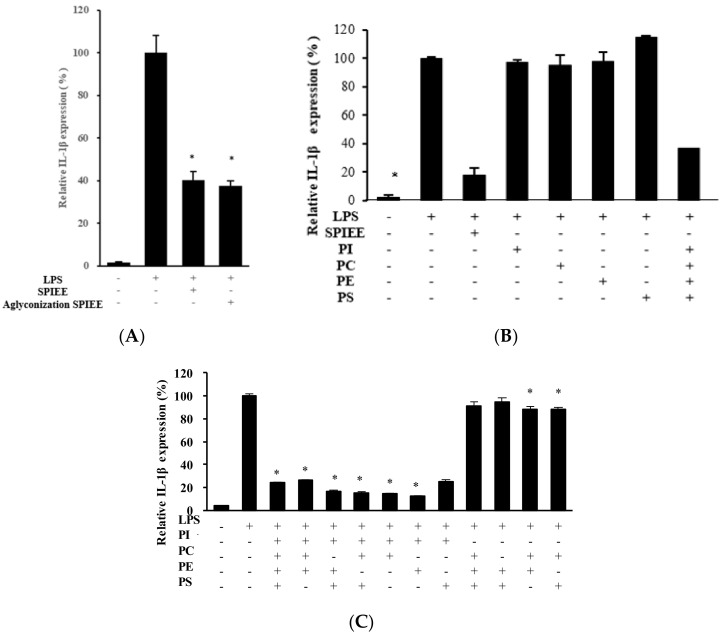
Effects of various substances on interleukin-1β (IL-1β) expression in macrophages following treatment with 100 ng/mL lipopolysaccharide (LPS). (**A**) Effects of SPIEE and aglyconized SPIEE. Concentration was 50 μg/mL in each experiment. (**B**) Effects of phosphatidylinositol (PI), phosphatidylcholine (PC), phosphatidylethanolamine (PE), phosphatidylserine (PS), and their mixture. The mixture was formulated at the same ratio as that found in SPIEE (Figure 10). Concentrations used were as follows: SPIEE, 50 μg/mL; PI, 3.5 μg/mL; PC, 5.5 μg/mL; PE, 2.0 μg/mL; and PS, 0.3 μg/mL. (**C**) Effect of each PL combination. After treatment with LPS and/or experimental samples for 6 h, IL-1β expression was determined using real-time PCR. Data are shown relative to the value in the LPS treatment group, which was set as 100%. * *p* < 0.05 vs. LPS treatment group. Data are shown relative to the value in the LPS treatment group, which was set as 100%. * *p* < 0.05 vs. LPS treatment group.

**Figure 10:**
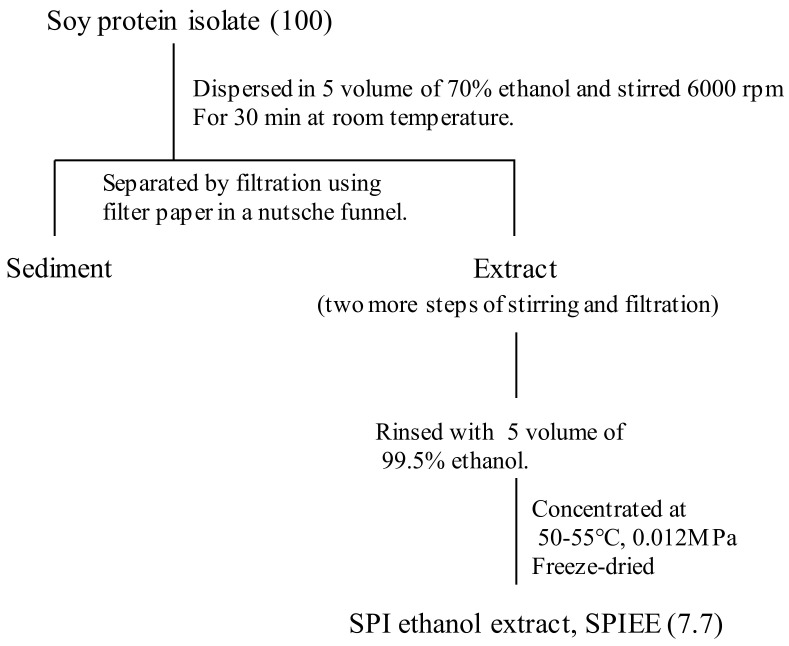
Schematic diagram for the preparation of soy protein isolate ethanol extract. Values in parentheses represent the yield of the SPIEE fraction.

The SPIEE was assessed for its anti-inflammatory effect using the macrophage cell line THP-1 derived from human monocytic leukemia. SPIEE consumption strongly inhibited IL-1β gene expression (Figure 4A). To clarify the contributions of the major SPIEE components, the anti-inflammatory effects of aglyconized SPIEE were investigated (refer to Table S1 for the isoflavone composition in SPIEE after aglyconization). SPIEE containing isoflavone glycosides and aglyconized SPIEE reduced the lipopolysaccharide (LPS)-induced IL-1β expression substantially, and their efficacy was almost the same (Figure 4A). Subsequently, the anti-inflammatory effects of PC, PI, PE, and PS, and a mixture of these PLs at the same ratio as that in SPIEE (Figure 10) were investigated. Although PL administration alone did not reduce the LPS-induced IL-1β expression, the mixture strikingly reduced IL-1β expression (Figure 4B). To identify the essential component(s), all possible PL combinations were assessed. As shown in Figure 4C, these combinations could clearly be classified as effective or non-effective, and PI was an essential component in other PLs. Thus, PI suppressed LPS-induced IL-1β expression in the presence of other PLs. 

**Figure 5:**
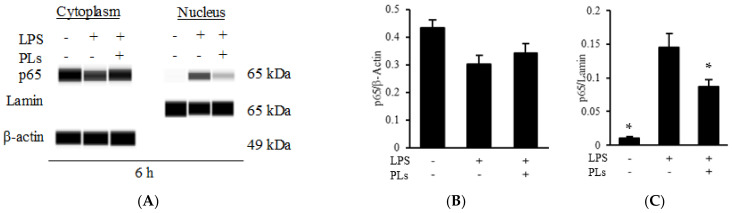

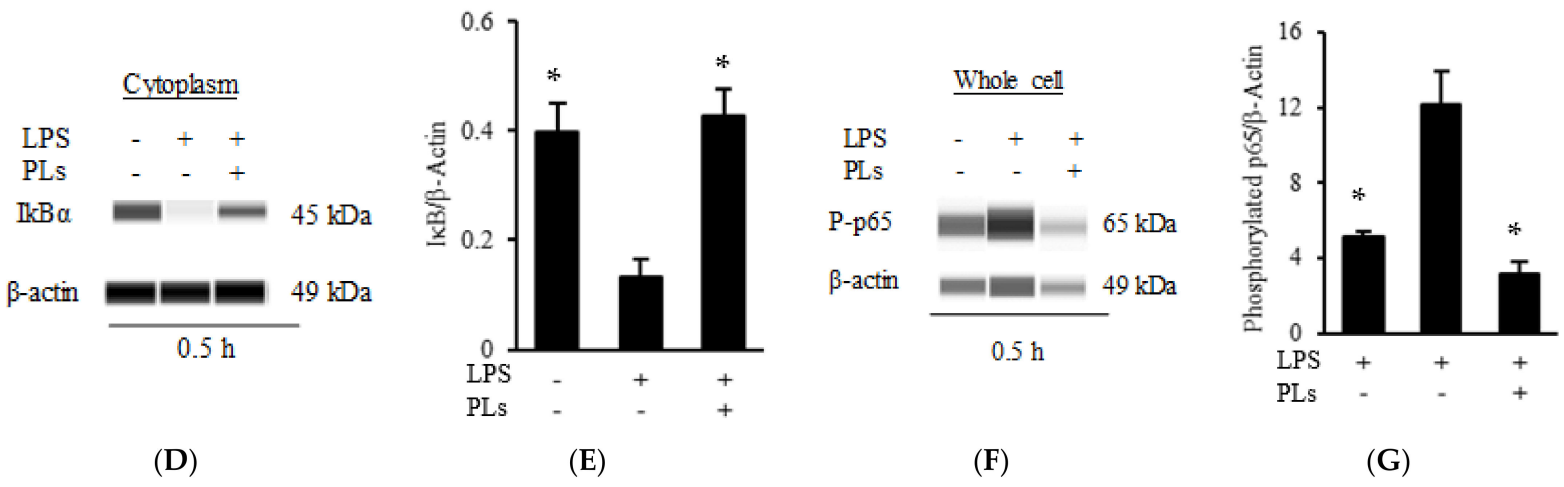
Effect of phospholipids (PLs) on nuclear factor-κB (NF-κB) signaling in macrophage cells treated with LPS. (**A**,**D**,**F**): Effects of PLs on the NF-κB pathway. THP-1 cells were treated with LPS or PLs. After 30 min and 6 h, p65, IκB, and phosphorylated p65 levels were detected by Western blotting; β-actin served as the loading control, and lamin served as the nuclear separation control. (**B**,**C**,**E**,**G**) Three Western blots in Figure 5A,D,F were statistically analyzed. Data are shown relative to the value in the LPS treatment group. * *p* < 0.05 vs. LPS treatment group.

Western blotting was performed to elucidate the effects of PLs on the nuclear factor kappa B (NF-κB) pathway in LPS-induced inflammation. THP-1–derived macrophages treated with PLs showed markedly suppressed nuclear p65 levels but high cytoplasmic p65 levels (Figure 5A–C). Lamin, a nuclear marker, was detected only in the nuclear fraction (Figure 5A). At 6 h after the onset of treatment, cytoplasmic p65 signals were detected at similar levels in all treatment groups, whereas nuclear p65 signals were strong in the LPS-treated group but relatively weak in the LPS + PL-treated group.

The effects of these treatments on the inhibitor κB (IκB) and p65 phosphorylation, which act upstream of NF-κB signaling, were also evaluated. Similar levels of the IκB signal were detected in the untreated and LPS + PL-treated groups, whereas those in the LPS-treated group were substantially weaker (Figure 5D,E). The level of the phosphorylated p65 signal increased with LPS treatment and decreased markedly with PL treatment (Figure 5F,G). Together, these findings indicated that PLs inhibit the degradation of IκB and p65 phosphorylation in the NF-κB pathway.

### 2.4. Genistein Suppresses MCP-1 Expression in Proximal Tubular Cells

**Figure 6:**
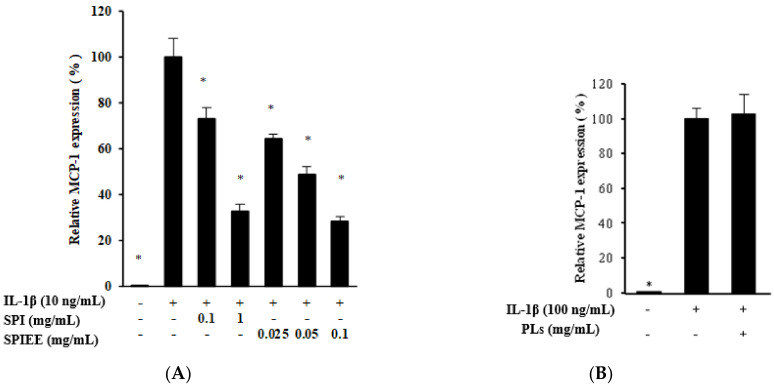

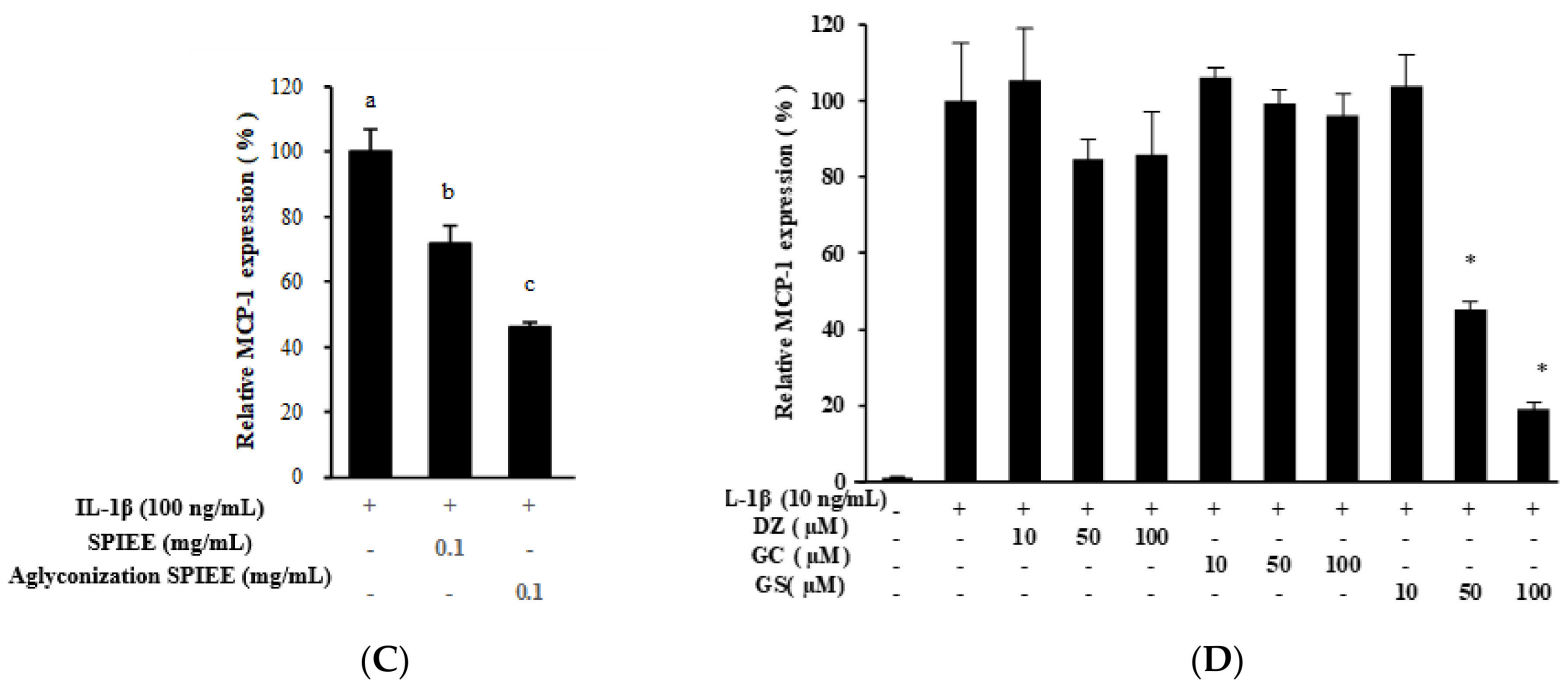
Effects of isoflavones and PLs on MCP-1 expression in proximal tubular cells following IL-1β treatment. Data are shown relative to the value in the IL-1β treatment group, which was set as 100% (**A**–**D**). * *p* < 0.05 vs. IL-1β treatment group. The results of multiple comparisons are represented by alphabets (a, b, c) on the column, indicating significant differences, *p* < 0.05 (**C**). Data indicate mean ± SEs, *n* = 3. A: SPIEE. B: PLs. (**C**) Aglyconization of SPIEE. The reduction of glycosides by aglyconization is shown in Table S1. (**D**) Isoflavone aglycones.

**Figure 7:**
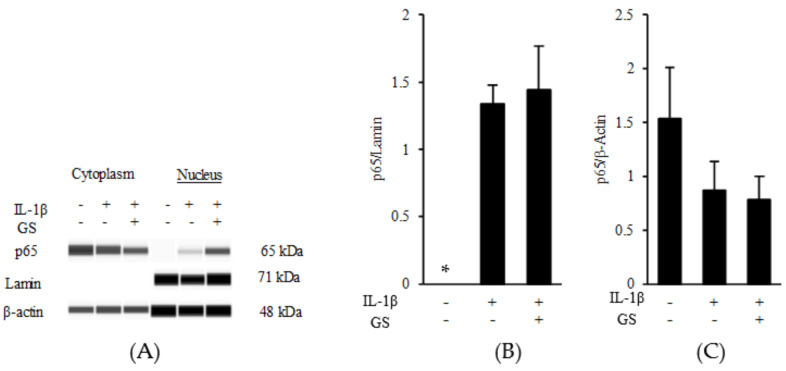
Effect of genistein (GS) on NF-κB signaling in proximal tubular cells treated with IL-1β. (**A**) Effect of genistein on the NF-κB pathway. NRK-52E cells were treated with IL-1β (10 ng/mL) or genistein (50 μM). After 2 h, p65 protein levels were analyzed by Western blotting. β-Actin served as the loading control, and lamin served as the nuclear separation control. (**B**,**C**) Statistical analysis of Figure 7A (*n* = 3). Data are shown relative to the value in the IL-1β treatment group. * *p* < 0.05 vs. IL-1β treatment group.

**Figure 8:**
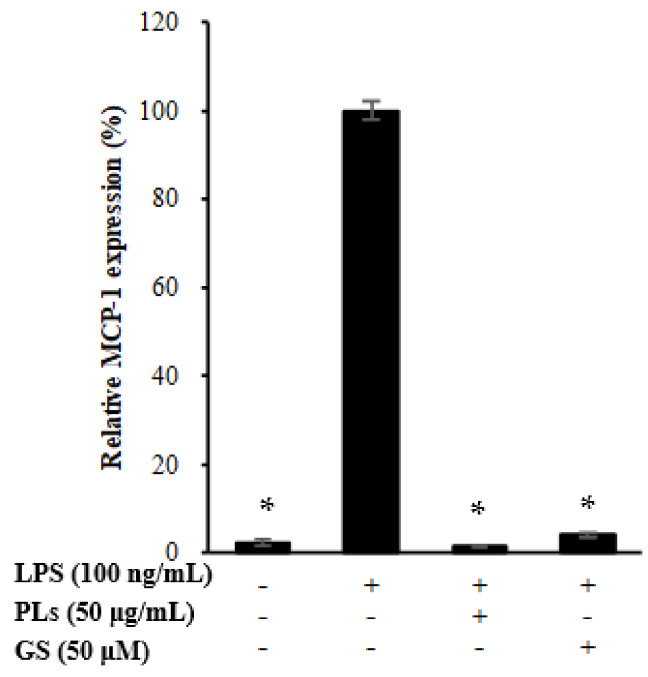
Effect of PLs and GS on MCP-1 expression in macrophage cells following LPS treatment. * *p* < 0.05 vs. LPS treatment group.

Next, whether SPIEE suppresses the expression of MCP-1, a chemotactic factor for macrophages, was investigated in vitro. In the renal proximal tubular cell line NRK-52E that epithelioid clone of normal rat kidney cells, SPIEE treatment significantly suppressed MCP-1 expression in a dose-dependent manner (Figure 6A). To clarify the SPIEE component(s) responsible for this effect, the effects of various components, including isoflavones (daidzin, glycitin, genistin, daidzein, glycitein, and genistein) and PLs, were evaluated separately. The PL mixture had no effect on MCP-1 expression; however, aglyconization of SPIEE had a stronger effect than that of SPIEE (Figure 6B,C). Evaluation of aglycones revealed that genistein attenuated MCP-1 expression in a dose-dependent manner (Figure 6D). Furthermore, whether genistein inhibits MCP-1 expression via the NF-κB pathway was investigated. As shown in Figure 7A–C, genistein did not suppress the nuclear translocation of p65, which is involved in the NF-κB pathway. Both genistein and PLs suppressed LPS-induced MCP-1 expression in THP-1 (Figure 8).

## 3. Discussion

Soy protein has been reported to inhibit the exacerbation of CKD [12]. In this study, SPIEE, the fat-soluble fraction of SPI, was found to be nearly as effective as SPI at inhibiting nephropathy and identified as the main component responsible for the renoprotective effect of SPI. This study used Zucker fatty rats as a model to evaluate CKD onset. The NAG values and body weight reported in previous studies based on Zucker rats suggested that these values were associated with CKD onset. In the present study, the NAG levels and weight of the Zucker fatty rats in the casein group were 0.6 U/day and 715.1 ± 22.2 g, respectively (Figure 1A and Table 2). Thus, the increase in NAG level and excess weight reported in previous studies was also observed in the present study [21,22].

While a mixture of PLs in SPIEE inhibited IL-1β expression in macrophages, the effect of isoflavones was the same regardless of whether glycosides or aglycones were present (Figure 4). Thus, the PLs could have potentially affected a reaction upstream of IκB breakdown and p65 phosphorylation in the NF-κB pathway. Further, PI was found to be essential for this inhibitory effect of PLs. Interestingly, the presence of both PI and at least one other PL component was required to inhibit IL-1β expression (Figure 4C), as this effect was not observed with PI alone (Figure 4C) or PI-free egg yolk lecithin (see Figure S1 and Table S2). This indicates that PI may be specifically involved in the control of IL-1β expression via regulation of the NF-κB pathway [23,24].

The reason why PLs are necessary for such an inhibitory effect of PI can be explained based on the inositol PL metabolic pathways. PI acts as a substrate for phospholipase C and promotes calcium release from the endoplasmic reticulum [25]. Furthermore, calcium-dependent arachidonic acid production via this metabolic pathway has been well documented. Arachidonic acid is non-specifically separated from PC, PE, and PI by phospholipase A2, which is activated in this pathway [26,27]. Lipoxin A4 is synthesized from arachidonic acid by 5-lipoxygenase (5-LOX) or 15-LOX, while Lipoxin A4 inhibits the activation of the NF-κB pathway in macrophages after LPS stimulation [28,29]. In addition, 5-LOX and 15-LOX are present in THP-1 cells [30,31,32]. In brief, PI induces arachidonic acid release from PC and PE, and the lipoxin A4 synthesized from arachidonic acid in THP-1 cells subsequently inhibits the NF-κB pathway. 

Based on the results of this study, a new hypothesis can be proposed based on the analysis of the anti-inflammatory effects of soya components in nephropathy. Namely, that the promotion of PI, calcium release, and arachidonic acid metabolism prevents renal deterioration via modulation of the NF-kB pathway. Future studies should focus on measurement of arachidonic acid levels in vitro to elucidate the PL metabolism.

According to a recent study on the intestinal microbiota, deterioration of the intestinal environment can contribute to CKD development, with inflammation caused by LPS originating from intestinal bacteria that are central to this process [33]. Higuchi et al. [34] reported that the antibacterial activity of rice peptide against *Escherichia coli*, which is an LPS producer, is responsible for the anti-inflammatory effect of rice protein. The inhibitory effect of SPIEE on the LPS-induced inflammatory pathway found in this study may be exploited for the treatment of kidney disease caused by deterioration of the intestinal environment.

Renal macrophage-mediated inflammation in the kidneys is also deeply involved in the development of renal disease [35]. Based on findings in a model of nephropathy progression, MCP-1 has been suggested as a diagnostic marker and therapeutic target [36]. The interaction between MCP-1, a chemokine also known as CCL2, and its receptor, C-C chemokine receptor (CCR2), is involved in macrophage infiltration [36,37]. The inhibition of macrophage infiltration and reduction in CCR2 expression induced by SPI intake strongly support the inhibition of macrophage migration via MCP-1 (Figure 3). In renal tubules, MCP-1 production is induced by various signals, and the signaling pathway induced by IL-1β and TNFα is particularly well known [38,39]. Decreased IL-1β expression in the kidneys following SPI and SPIEE intake may be an important factor in preventing kidney disease progression.

**Figure 9:**
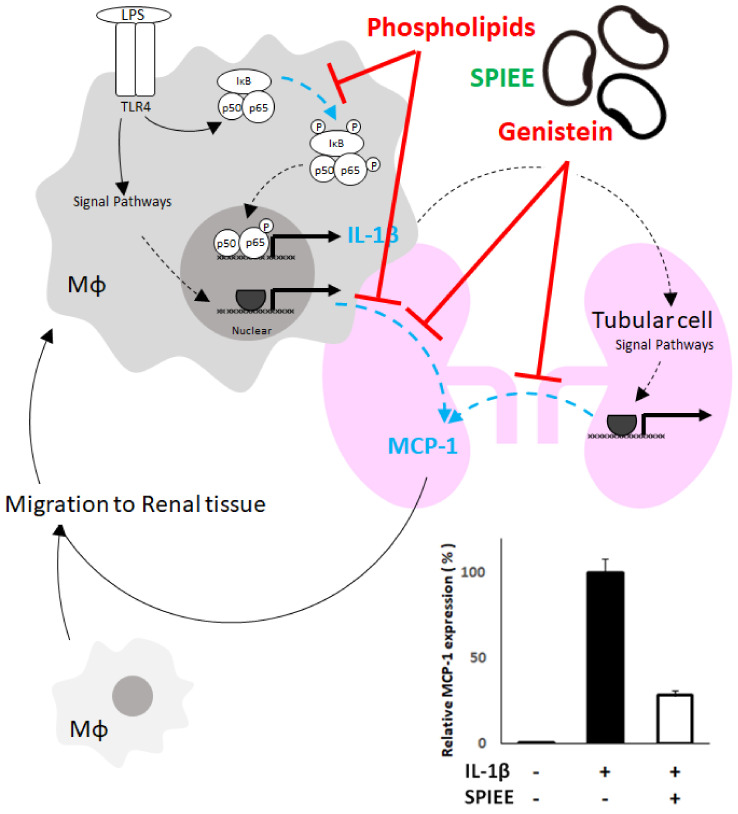
Putative roles of PLs and GS in kidney disease. Schematic illustration of the mechanisms of PLs and GS in renal inflammation. SPIEE contains PLs and GS. PLs suppress IL-1β expression by inhibiting the NF-κB pathway in Mφ and suppress MCP-1 expression in Mφ, and GS suppresses MCP-1 expression in renal tubules and Mφ. Suppression of the renal inflammatory cycle through these actions is considered to contribute to the slowing down of nephropathy progression.

The in vivo beneficial effects of SPIEE ingestion are discussed based on the hypothesis that macrophages infiltrating the kidney produce IL-1β, which stimulates renal tubular cells to produce MCP-1 and promotes macrophage infiltration via a feedback mechanism. Furthermore, as macrophages produce MCP-1, they must also induce autocrine infiltration. Screening of the active components in SPIEE revealed that genistein inhibited MCP-1 expression in renal tubule cells and macrophages, while PLs inhibited MCP-1 and IL-1β expression in macrophages (Figure 9). A signaling cascade that leads to MCP-1 induction is activated by reactive oxygen species (ROS) [40]. In a streptozotocin-induced type I diabetes animal model, genistein inhibited MCP-1 expression by suppressing ROS activity in renal tubules [40]. However, in the present study, the ROS pathway was excluded as an inhibitory target of genistein as renal tubule cells are directly stimulated by IL-1β, independent of ROS. In a previous study using mesangial cells, IL-1β–induced MCP-1 expression was suppressed upon inhibition of the NF-κB pathway [41]. In the present study, nuclear translocation of p65, a component of IL-1β-induced NF-κB signaling, was also observed in NRK52E cells; however, genistein did not inhibit p65 nuclear translocation. As a presumably different signaling cascade, the inhibition of MCP-1 expression by genistein in renal tubules may be regulated farther downstream of the nuclear translocation of p65. IL-1β-induced MCP-1 expression is also observed in the JNK/AP-1 pathway [42]. LPS-induced MCP-1 expression is suppressed by inhibitors of p38, JNK, and MAPK [43,44,45]. Thus, MCP-1 may act through a pathway other than the NF-κB signaling cascade. Furthermore, the mechanism by which genistein suppresses LPS-induced MCP-1 expression in macrophages may also be different from that involving NF-κB. Further research is thus needed to identify the mechanism of action of genistein.

Genistein and PLs, which were identified as key components in SPIEE, need to reach the kidneys to exert their effects. Whether they reach the macrophages and proximal tubular cells should be verified in future studies. However, orally ingested isoflavones and PLs have been reported to remain stable in the blood for 72 and 100 h, respectively, without being degraded or metabolized [46,47]. Furthermore, isoflavones are detectable in urine and are considered to reach the kidneys [48]. Since PLs are known to exert their functions in various organs, they are thought to circulate in the body and reach the kidneys while maintaining their bioactivity [49]. Saponins are a part of SPIEE but are mostly metabolized by intestinal bacteria in the large intestine; therefore, they were excluded as main contributors of the biological actions of SPIEE [50,51,52].

SPI was more effective at inhibiting protein excretion into the urine than SPIEE, possibly because of the presence of peptides in SPI (Figure 1B). Peptides, which are a product of soy protein hydrolysis, reduce inflammation of the colon and ileum in a porcine model with dextran sodium sulfate-induced colitis [53] and suppress IL-1β-induced matrix-degrading enzymes in human articular chondrocytes [54]. Whether the anti-inflammatory effect of peptides also contributes to CKD prevention remains to be investigated.

Some of the inhibitory effects of either genistein or PI on CKD progression are due to anti-inflammatory action, and the cause of macrophage migration promotion remains to be clarified in future studies. As chronic inflammation caused by macrophages is common in lifestyle-related diseases [55], strategies for suppressing the inflammatory cascade using PI as an active ingredient may be applied to prevent inflammation in other organs and diseases.

## 4. Materials and Methods

### 4.1. Preparation of SPIEE and Fractionation

Commercially used SPI (Fuji-pro; Fuji Oil, Osaka, Japan) was used for the preparation of SPIEE (Figure 10); 1000 g of SPI was dispersed in 5 L 70% ethanol and stirred with a homomixer at 6000 rpm for 30 min. The 70% ethanol-soluble fraction was then separated by filtration using filter paper (No. 1, 330 mm; Toyo Roshi Kaisha, Tokyo, Japan) in a Nutsche funnel. The filtered residue was dispersed in 5 L 70% ethanol as described above, followed by two more steps of stirring and filtration. The insoluble fraction was rinsed with 99.5% ethanol. SPIEE was prepared from the ethanol-soluble fractions by freeze-drying after evaporation of the ethanol. The yield of SPIEE from the initial SPI volume was 77 g. The aglyconization of SPIEE was carried out using β-glucosidase (Aromase^TM^ H2; Amano enzyme, Aichi, Japan). The reactants were incubated overnight, at 60 °C in 10 mM acetate buffer (pH 5.0) at 1% substrate and 1% enzyme concentrations. The enzyme in the mix was heat inactivated by incubation at 100 °C for 20 min. The SPIEE was extracted with 70% ethanol, according to the method described above.

### 4.2. Detection of Isoflavonoid Species and PLs

By a modification of the methods of kudou et al. [56], high-performance liquid chromatography (HPLC) was performed to quantify isoflavones in the SPIEE fraction. In brief, test materials were extracted three times by agitation with 10 volumes of 70% ethanol for 30 min at 25 °C. After centrifugation, isoflavones in the supernatant were analyzed by quantitative HPLC on a YMC-pack ODS-AM-303 column (250 × 4.6 min) using a linear gradient of acetonitrile (water/acetonitrile, 85:15–65:35 *v/v*) containing constant 0.1% acetic acid for 50 min at 35 °C; absorbance was measured at 254 nm. The solvent flow rate was 1 mL/min. The amount of each isoflavone was determined as the aglycon equivalent, using the following formula. Area × slope × coefficient of correction (molecular weight of aglycon/molecular weight of glycoside). PLs were quantified using molybdenum blue absorptiometry after TLC at the Japan Food Research Laboratories (Osaka, Japan).

### 4.3. Animals and Animal Experimental Design

In vivo experiments were performed according to the protocols reported by Trujillo et al. [22], with some modifications. In total, 15 five-week-old Zucker fa/fa (fatty) male rats were purchased from Charles River Japan (Tokyo, Japan) and housed individually in stainless steel cages under controlled conditions (23° ± 1 °C temperature; 55 ± 5% humidity; and 12/12 h light/dark cycles). The rats were acclimated to commercial chow (CRF-1; Oriental Yeast, Tokyo, Japan) for 1 week prior to experimentation. Vitamin-free casein (casein; Oriental Yeast) and SPI were provided as dietary protein sources (20% crude protein of the experimental diets). After acclimation, the rats were divided into three groups with near-equal average body weights and urinary protein excretion levels. The casein diet was used in the control group, the second group was fed an SPI diet, and the third group was fed a casein + SPIEE diet. The experimental diets were based on an adjusted AIN-93G formula to ensure equal protein administration for all groups (Table 3). In this study, water and the experimental diets were provided ad libitum. Food intake and body weight were recorded daily and bi-weekly, respectively. The rats were placed in metabolic cages on the 1st day of weeks 2, 4, 8, 12, and 16 after experimental feeding to collect 24 h total urine samples. The urine samples were centrifuged at 3000× *g* at 4 °C for 10 min, and supernatants were stored at −80 °C until analysis.

After 16 weeks of experimental diet, blood was collected from the abdominal aorta in a non-fasted state using a heparinized syringe—performed under isoflurane anesthesia administration with a small animal gas anesthesia system to minimize pain (Dainippon Sumitomo Pharma, Tokyo, Japan). Kidneys were collected and stored at −80 °C.

### 4.4. Urinalysis

Urinary protein excretion level was determined using the Coomassie brilliant blue G-250 method (Tonein-TP; Otsuka Pharmaceutical, Tokushima, Japan) [57]. Urinary *N*-acetyl-β-d-glucosaminidase (NAG) levels were measured using a colorimetric method (NAG Test Shionogi; Shionogi, Osaka, Japan) [58]. 

### 4.5. Histochemical Staining

Kidneys were fixed in a 10% formalin-phosphate buffer (FUJIFILM Wako Pure Chemical, Tokyo, Japan), embedded in paraffin, and sectioned at 5 µm thickness. The sections were subjected to periodic acid-Schiff staining to determine the status of mesangial matrix deposition and renal fibrosis. Renal inflammation was evaluated by anti-CD68 antibody clone ED1 (ED-1) staining (a technical service provided by Sapporo General Pathology Laboratory, Sapporo, Japan). The staining intensity of ED-1-positive cells in the interstitium of the tubules was investigated by optical microscopy at 20× magnification and scored as follows: 0, absent; 1, weak; 2, moderate; 3, severe; and 4, very severe. For each individual, 15 fields of view were scored, and the average for each group was reported as the “positive cell score.”

### 4.6. Cell Culture and Treatments

Purified isoflavones (daidzein, glycitein, and genistein) were purchased from Nagara Science (Gifu, Japan) and purified PLs [phosphatidylserine (PS), phosphatidylinositol (PI), phosphatidylcholine (PC), and phosphatidylethanolamine (PE) were purchased from Avanti Polar Lipids (Alabaster, AL, USA). l-α-phosphatidylcholine (from soybean; Sigma-Aldrich Japan, Tokyo, Japan) was used as crude PL. The isoflavones and the l-α-phosphatidylcholine were dissolved in 70% EtOH and added to the medium at a 200-fold dilution. The PLs were each dissolved in chloroform at 5 mg/mL and mixed at the ratio presented for the SPIEE to prepare a PL mixture. The mixture was further dissolved in 70% EtOH and added to the medium at a 200-fold dilution.

The immortalized rat tubular epithelial cell line NRK-52E and the human monocyte cell line THP-1 were obtained from the American Type Culture Collection (Manassas, VA, USA). The NRK-52E cells were maintained in Dulbecco’s modified Eagle’s medium (Thermo Fisher SCIENTIFIC, Tokyo, Japan) supplemented with 5% fetal bovine serum (FBS), and THP-1 cells were cultured in Roswell Park Memorial Institute medium (Thermo Fisher SCIENTIFIC) supplemented with 10% FBS. All cells were cultured at 37 °C in humidified air containing 5% CO_2_. 

Subconfluent NRK-52E cells were cultured in serum-free medium for 24 h; then, the medium was replaced with a serum-free medium containing 10 ng/mL IL-1β and/or experimental samples, followed by incubation for 2 h. THP-1 cells were differentiated into macrophages and treated with phorbol 12-myristate 13-acetate (PMA) for 48 h. Then, the medium was changed to the one containing 100 ng/mL lipopolysaccharide (LPS) and/or experimental samples, followed by incubation for 6 h. IL-β, PMA and LPS were purchased from Sigma-Aldrich Japan.

### 4.7. Gene Expression Analysis

Total RNA was isolated from rat kidney tissues and cultured cells using ISOGEN (Nippon Gene, Tokyo, Japan) and purified using RNeasy spin columns (RNeasy Mini Kit; Qiagen, Hilden, Germany) according to the manufacturers’ instructions. The total RNA was reverse-transcribed into cDNA using a PrimeScript™ RT reagent kit (Takara Bio, Tokyo, Japan).

Real-time semiquantitative PCRs were run using 25 ng cDNA and 50 pM gene-specific primers (TaqMan^®^ Gene Expression Assays; Applied Biosystems Japan, Tokyo, Japan) in a StepOnePlus™ sequence detection system (Applied Biosystems Japan). The samples were incubated at 95 °C for 10 min for initial denaturation, followed by 40 cycles at 95 °C for 30 s, 60 °C for 30 s, and 72 °C for 20 s.

Specific primers (sourced by TaqMan^®^ Gene Expression Assays) targeting the following rat genes were used: Col1a1, encoding type I collagen (Rn01463848), TGF-β (Rn00572010), MCP-1 (Rn00580555), IL-1β (Rn00580432, Hs01555410), PAI-1 (Rn01481341), CCR2 (Rn01637698), and GAPDH (Rn01775763, Hs002759991). GAPDH mRNA expression was determined for the normalization of target gene expressions. For the quantification of gene expression, the comparative cycle threshold method was applied using the ΔΔCt arithmetic formula.

### 4.8. Western Blotting

Nuclear and cytosolic proteins from NRK-52E and THP-1 cells were separated using a Cytoplasmic & Nuclear Protein Extraction Kit (101Bio, Mountain View, CA, USA) according to the manufacturer’s instructions. Western blot analysis was performed with a ProteinSimple^®^ WES System (ProteinSimple Japan K.K., Tokyo, Japan) according to the manufacturer’s instructions. Primary antibodies against β-actin (MAB8929; R&D Systems, Minneapolis, MN, USA), IκB-α (#6A920; Novus Biologicals, Centennial, CO, USA), NF-κB-p65 (#8242; Cell Signaling Technology, Danvers, MA, USA), Phospho-NF-κB p65 (#3033; Cell Signaling Technology), and lamin B1 (ab16048; Abcam, Cambridge, MA, USA) were used.

### 4.9. Statistical Analysis

Data are expressed as the mean ± standard error (SE) of the mean. Dunnett’s test was used for comparisons with the control group. Multiple comparisons were performed using Tukey’s test. Ordinal scale data were analyzed using a Kruskal–Wallis test. *p* < 0.05 was considered statistically significant. Statistical analyses were performed using SPSS software (12.0J; SPSS, Chicago, IL, USA).

## 5. Conclusions

In the present study, SPIEE was found to exert renoprotective effects. As demonstrated by cell assays, genistein, a component of SPIEE, suppressed IL-1β-induced MCP-1 expression in tubular cells and LPS-induced MCP-1 expression in macrophage cells. Moreover, soybean PLs suppressed LPS-induced IL-1β and MCP-1 expression in macrophages. Genistein and the PLs contained in soybean contribute to the inhibition of kidney disease exacerbation by inhibiting kidney inflammation. The mechanism involves the suppression of IL-1β expression by inhibiting the phosphorylation of IκB and p65, components of the NF-κB pathway, in macrophages. PI was observed to be essential for such activity. Further studies of the anti-inflammatory effects of PLs, including studies of arachidonic acid-derived mediators, are necessary. This study proposes anti-inflammatory effects as the potential mechanisms underlying the inhibitory effects of soybean on nephropathy. These findings have implications for the promotion of daily dietary soy intake and supplementation for nephropathy patients.

## Figures and Tables

**Table 1:** The composition of phospholipids and isoflavones in SPIEE.

PC	10.9
PE	3.94
PI	7.04
PS	0.64
Daidzein	1.31
Glycitein	0.23
Genistein	1.98
	(g/100g)

The value for each isoflavone indicates the aglycon equivalent. PC, phosphatidylcholine; PE, phosphatidylethanolamine; PI, phosphatidylinositol; PS, phosphatidylserine.

**Table 2:** Growth parameters in fatty rats after 16 weeks of experimental diet consumption.

		Cas	SPI	SPIEE	*p*-Values (vs. Cas)
	(*n*)	5	5	5	(SPI, SPIEE)
Initial weight (g)		313.2 ± 6.0	314.3 ± 4.4	314.3 ± 5.1	0.983, 0.983
Final weight (g)		715.1 ± 22.2	698.6 ± 9.0	733.4 ± 16.4	0.715, 0.663
Food intake (g/day)		25.4 ± 0.9	23.4 ± 0.5	24.6 ± 0.7	0.109, 0.637
Food efficiency ratio		0.139 ± 0.005	0.145 ± 0.005	0.150 ±0.003	0.548, 0.16

The values represent mean ± SEs. Cas, casein; SPI, soy protein isolate; SPIEE, SPI ethanol extract.

**Table 3:** Compositions of the experimental diets.

	Casein	SPI	SPIEE
Protein source			
Casein ^§^	227		227
SPI ^†^		234	
SPIEE			18.1
Soybean oil ^‡^	70	70	70
Cornstarch	373	367	355
Sucrose	100	100	100
Dextrinized cornstarch	132	132	132
Cellulose powder	50	50	50
Mineral mixture ^║^	35	35	35
Vitamin mixture ^║^	10	10	10
Choline bitartrate	3	3	3
Total (g)	1000	1000	1000

The amount of SPIEE was calculated from the ratio of extraction as described in the Methods section. ^§^ Crude protein content: 88.1% (Oriental Yeast, Tokyo, Japan). ^†^ Crude protein content: 85.5% (Fuji Oil, Osaka, Japan). ^‡^ The soybean oil contains 0.02% *t*-butylhydroquinone. ^║^ AIN-93G mixtures (Oriental Yeast). Abbreviations: SPI, soy protein isolate; SPIEE, SPI ethanol extract.

## Data Availability

The data presented in this study are available on request from the corresponding author. The data are not publicly available due to have not setup a public archive platform for data sharing.

## Supplementary Materials

The following supporting information can be downloaded at: https://www.mdpi.com/article/10.3390/metabo12040330/s1. Table S1: Isoflavone composition in non-enzymatically treated SPIEE and aglyconized SPIEE; Table S2: Phospholipids in soy and egg lecithin; Figure S1: Comparison of the inhibitory effects of soy and egg lecithin on IL-1β expression in THP-1 cells; Figure S2: Chromatogram of the separation of isoflavones.

## References

[1] Go A.S., Chertow G.M., Fan D., McCulloch C.E., Hsu C.-Y. (2004). Chronic kidney disease and the risks of death, cardiovascular events, and hospitalization. N. Engl. J. Med..

[2] Zhang X., Lerman L.O. (2017). The metabolic syndrome and chronic kidney disease. Transl. Res..

[3] Wahba I.M., Mak R.H. (2007). Obesity and obesity-initiated metabolic syndrome: Mechanistic links to chronic kidney disease. Clin. J. Am. Soc. Nephrol..

[4] Lu L., Huang Y.-F., Wang M.-Q., Chen D.-X., Wan H., Wei L.-B., Xiao W. (2017). Dietary fiber intake is associated with chronic kidney disease (CKD) progression and cardiovascular risk, but not protein nutritional status, in adults with CKD. Asia Pac. J. Clin. Nutr..

[5] Asghari G., Momenan M., Yuzbashian E., Mirmiran P., Azizi F. (2018). Dietary pattern and incidence of chronic kidney disease among adults: A population-based study. Nutr. Metab..

[6] Gai Z., Wang T., Visentin M., Kullak-Ublick G., Fu X., Wang Z. (2019). Lipid accumulation and chronic kidney disease. Nutrients.

[7] Kelly J., Palmer S.C., Wai S.N., Ruospo M., Carrero J.-J., Campbell K., Strippoli G.F.M. (2016). Healthy dietary patterns and risk of mortality and ESRD in CKD: A meta-analysis of cohort studies. Clin. J. Am. Soc. Nephrol..

[8] Caponio G.R., Wang D.Q.-H., Di Ciaula A., De Angelis M., Portincasa P. (2020). Regulation of cholesterol metabolism by bioactive components of soy proteins: Novel translational evidence. Int. J. Mol. Sci..

[9] Kohno M., Hirotsuka M., Kito M., Matsuzawa Y. (2006). Decreases in serum triacylglycerol and visceral fat mediated by dietary soybean.BETA.-conglycinin. J. Atheroscler. Thromb..

[10] Tachibana N., Yamashita Y., Nagata M., Wanezaki S., Ashida H., Horio F., Kohno M. (2014). Soy β-conglycinin improves glucose uptake in skeletal muscle and ameliorates hepatic insulin resistance in Goto-Kakizaki rats. Nutr. Res..

[11] McGraw N.J., Krul E.S., Grunz-Borgmann E., Parrish A.R. (2016). Soy-based renoprotection. World J. Nephrol..

[12] Kafeshani M., Rafieian-Kopaei M., Beigrezaei S., Nasri H. (2017). Soy protein and chronic kidney disease: An updated review. Int. J. Prev. Med..

[13] Azadbakht L., Atabak S., Esmaillzadeh A. (2008). Soy protein intake, cardiorenal indices, and c-reactive protein in type 2 diabetes with nephropathy. Diabetes Care.

[14] Jheng H.-F., Hirotsuka M., Goto T., Shibata M., Matsumura Y., Kawada T. (2017). Dietary low-fat soy milk powder retards diabetic nephropathy progression via inhibition of renal fibrosis and renal inflammation. Mol. Nutr. Food Res..

[15] Shikata K., Makino H. (2013). Microinflammation in the pathogenesis of diabetic nephropathy. J. Diabetes Investig..

[16] Black L.M., Lever J.M., Agarwal A. (2019). Renal inflammation and fibrosis: A double-edged sword. J. Histochem. Cytochem..

[17] Asanoma M., Tachibana N., Hirotsuka M., Kohno M., Watanabe Y. (2012). Effects of soy protein isolate feeding on severe kidney damage in DOCA salt-treated obese zucker rats. J. Agric. Food Chem..

[18] Palanisamy N., Kannappan S., Anuradha C.V. (2011). Genistein modulates NF-κB-associated renal inflammation, fibrosis and podocyte abnormalities in fructose-fed rats. Eur. J. Pharmacol..

[19] Palanisamy N., Viswanathan P., Anuradha C.V. (2008). Effect of genistein, a soy isof lavone, on whole body insulin sensitivity and renal damage induced by a high-fructose diet. Ren. Fail..

[20] Ogborn M.R., Nitschmann E., Bankovic-Calic N., Weiler H.A., Aukema H.M. (2010). Dietary soy protein benefit in experimental kidney disease is preserved after isoflavone depletion of diet. Exp. Biol. Med..

[21] Sato N., Kaneko M., Tamura M., Kurumatani H. (2010). The prostacyclin analog beraprost sodium ameliorates characteristics of metabolic syndrome in obese zucker (fatty) rats. Diabetes.

[22] Trujillo J., Ramírez V., Pérez J., Torre-Villalvazo I., Torres N., Tovar A.R., Muñoz R.M., Uribe N., Gamba G., Bobadilla N.A. (2005). Renal protection by a soy diet in obese Zucker rats is associated with restoration of nitric oxide generation. Am. J. Physiol. Physiol..

[23] Liu T., Zhang L., Joo D., Sun S.-C. (2017). NF-κB signaling in inflammation. Signal Transduct. Target. Ther..

[24] Pahl H.L. (1999). Activators and target genes of Rel/NF-kappaB transcription factors. Oncogene.

[25] Balla T., Szentpetery Z., Kim Y.J. (2009). Phosphoinositide signaling: New tools and insights. Physiology.

[26] Tadaomi T., Ishitoya J., Homma Y., Kato M., Nagai Y. (1985). Role of enhanced inositol phospholipid metabolism in neutrophil activation. Biochem. Pharmacol..

[27] Takenawa T., Homma Y., Nagai Y. (1983). Role of Ca^2+^ in phosphatidylinositol response and arachidonic acid release in formylated tripeptide- or Ca^2+^ ionophore A23187-stimulated guinea pig neutrophils. J. Immunol..

[28] Huang Y.-H., Wang H.-M., Cai Z.-Y., Xu F.-Y., Zhou X.-Y. (2014). Lipoxin A4 Inhibits NF-κB activation and Cell Cycle Progression in RAW264.7 Cells. Inflammation.

[29] Kure I., Nishiumi S., Nishitani Y., Tanoue T., Ishida T., Mizuno M., Fujita T., Kutsumi H., Arita M., Azuma T. (2009). Lipoxin A4Reduces lipopolysaccharide-induced inflammation in macrophages and intestinal epithelial cells through inhibition of nuclear factor-kappaB activation. J. Pharmacol. Exp. Ther..

[30] Serio K.J., Johns S.C., Luo L., Hodulik C.R., Bigby T.D. (2003). Lipopolysaccharide down-regulates the leukotriene C4Synthase gene in the monocyte-like cell line, THP-1. J. Immunol..

[31] Riddick C.A., Ring W.L., Baker J.R., Hodulik C.R., Bigby T.D. (1997). Dexamethasone increases expression of 5-lipoxygenase and its activating protein in human monocytes and THP-1 Cells. Eur. J. Biochem..

[32] Weibel G.L., Joshi M.R., Wei C., Bates S.R., Blair I.A., Rothblat G.H. (2009). 15(S)-Lipoxygenase-1 associates with neutral lipid droplets in macrophage foam cells: Evidence of lipid droplet metabolism. J. Lipid Res..

[33] Mahmoodpoor F., Rahbar Saadat Y., Barzegari A., Ardalan M., Zununi Vahed S. (2017). The impact of gut microbiota on kidney function and pathogenesis. Biomed. Pharmacother..

[34] Higuchi Y., Hosojima M., Kabasawa H., Kuwahara S., Goto S., Toba K., Kaseda R., Tanaka T., Kitamura N., Takihara H. (2019). Rice endosperm protein administration to juvenile mice regulates gut microbiota and suppresses the development of high-fat diet-induced obesity and related disorders in adulthood. Nutrients.

[35] Cao Q., Harris D.C.H., Wang Y. (2015). Macrophages in kidney injury, inflammation, and fibrosis. Physiology.

[36] Kitagawa K., Wada T., Furuichi K., Hashimoto H., Ishiwata Y., Asano M., Takeya M., Kuziel W.A., Matsushima K., Mukaida N. (2004). Blockade of CCR2 ameliorates progressive fibrosis in kidney. Am. J. Pathol..

[37] Wada T., Furuichi K., Sakai N., Iwata Y., Kitagawa K., Ishida Y., Kondo T., Hashimoto H., Ishiwata Y., Mukaida N. (2004). Gene therapy via blockade of monocyte chemoattractant protein-1 for renal fibrosis. J. Am. Soc. Nephrol..

[38] Blanchard O., Stepanovska B., Starck M., Erhardt M., Römer I., Zu Heringdorf D.M., Pfeilschifter J., Zangemeister-Wittke U., Huwiler A. (2018). Downregulation of the S1P transporter spinster homology protein 2 (Spns2) exerts an anti-fibrotic and anti-inflammatory effect in human renal proximal tubular epithelial cells. Int. J. Mol. Sci..

[39] Sarközi R., Corazza U., Osterkamp J.-P., Pirklbauer M., Mayer G., Schramek H. (2015). Synergistic induction of CCL2/MCP-1 expression driven by oncostatin M and IL-1β in human proximal tubular cells depends on STAT3 and p65 NFκB/RelA. Physiol. Rep..

[40] Sung M.J., Kim D.H., Jung Y.J., Kang K.P., Lee A.S., Lee S., Kim W., Davaatseren M., Hwang J.-T., Kim H.-J. (2008). Genistein protects the kidney from cisplatin-induced injury. Kidney Int..

[41] Rovin B.H., Lu L., Cosio A. (2001). Cyclopentenone prostaglandins inhibit cytokine-induced nf-kappab activation and chemokine production by human mesangial cells. J. Am. Soc. Nephrol..

[42] Lee M.-J., Yang C.W., Jin D.C., Chang Y.S., Bang B.K., Kim Y.-S. (2003). Bone morphogenetic protein-7 Inhibits constitutive and interleukin-1β-induced monocyte chemoattractant protein-1 expression in human mesangial cells: Role for JNK/AP-1 pathway. J. Immunol..

[43] Wang Y.-C., Hsieh C.-C., Kuo H.-F., Tsai M.-K., Yang S.-N., Kuo C.-H., Lee M.-S., Hung C.-H. (2014). Effect of vitamin D3 on monocyte chemoattractant protein 1 production in monocytes and macrophages. Acta Cardiol. Sin..

[44] Nomura J., Busso N., Ives A., Tsujimoto S., Tamura M., So A., Yamanaka Y. (2013). Febuxostat, an inhibitor of xanthine oxidase, suppresses lipopolysaccharide-induced MCP-1 production via MAPK phosphatase-1-mediated inactivation of JNK. PLoS ONE.

[45] Guha M., O’Connell M.A., Pawlinski R., Hollis A., McGovern P., Yan S.F., Stern D., Mackman N. (2001). Lipopolysaccharide activation of the MEK-ERK1/2 pathway in human monocytic cells mediates tissue factor and tumor necrosis factor alpha expression by inducing Elk-1 phosphorylation and Egr-1 expression. Blood.

[46] Setchell K.D.R., Faughnan M.S., Avades T., Zimmer-Nechemias L., Brown N.M., Wolfe B.E., Brashear W.T., Desai P., Oldfield M.F., Botting N.P. (2003). Comparing the pharmacokinetics of daidzein and genistein with the use of ^13^C-labeled tracers in premenopausal women. Am. J. Clin. Nutr..

[47] Zierenberg O., Grundy S.M. (1982). Intestinal absorption of polyene phosphatidylcholine in man. J. Lipid Res..

[48] Manach C., Williamson G., Morand C., Scalbert A., Rémésy C. (2005). Bioavailability and bioefficacy of polyphenols in humans. I. Review of 97 bioavailability studies. Am. J. Clin. Nutr..

[49] Küllenberg D., Taylor L.A., Schneider M., Massing U. (2012). Health effects of dietary phospholipids. Lipids Health Dis..

[50] Fukui K., Tachibana N., Wanezaki S., Tsuzaki S., Takamatsu K., Yamamoto T., Hashimoto Y., Shimoda T. (2002). Isoflavone-free soy protein prepared by column chromatography reduces plasma cholesterol in rats. J. Agric. Food Chem..

[51] Hu J., Zheng Y.L., Hyde W., Hendrich S., Murphy P.A. (2004). Human fecal metabolism of soyasaponin I. J. Agric. Food Chem..

[52] Hu J., Reddy M.B., Hendrich S., Murphy P.A. (2004). Soyasaponin I and Sapongenol B have limited absorption by Caco-2 intestinal cells and limited bioavailability in women. J. Nutr..

[53] Young D., Ibuki M., Nakamori T., Fan M., Mine Y. (2011). Soy-Derived Di- and tripeptides alleviate colon and ileum inflammation in pigs with dextran sodium sulfate-induced colitis. J. Nutr..

[54] Arito M., Mitsui H., Kurokawa M.S., Yudoh K., Kamada T., Niki H., Kato T. (2016). Effects of soy peptides on IL-1β-induced matrix-degrading enzymes in human articular chondrocytes. Integr. Mol. Med..

[55] Dandona P., Aljada A., Bandyopadhyay A. (2004). Inflammation: The link between insulin resistance, obesity and diabetes. Trends Immunol..

[56] Kudou S., Fleury Y., Welti D., Magnolato D., Uchida T., Kitamura K., Okubo K. (1991). Malonyl isoflavone glycosides in soybean seeds ( Glycine max Merrill). Agric. Biol. Chem..

[57] Lott J.A., Stephan V.A., Pritchard K.A. (1983). Evaluation of the coomassie brilliant blue G-250 method for urinary protein. Clin. Chem..

[58] Noto A., Ogawa Y., Mori S., Yoshioka M., Kitakaze T., Hori T., Nakamura M., Miyake T. (1983). Simple, rapid spectrophotometry of urinary N-acetyl-beta-D-glucosaminidase, with use of a new chromogenic substrate. Clin. Chem..

