# Supplementary material for: Soy Phospholipids Exert a Renoprotective Effect by Inhibiting the Nuclear Factor Kappa B Pathway in Macrophages
Source: Metabolites. 2022 Apr 6;12(4):330. doi: 10.3390/metabo12040330 (PMC9031346; doi:10.3390/metabo12040330)
Supplement: Supplementary file 1 [file metabolites-12-00330-s001.zip › metabolites-1654677-supplementary.pdf]

metabolites

MDPI

Article

Soy Phospholipids Exert a Renoprotective Effect by Inhibiting the Nuclear Factor Kappa B Pathway in Macrophages

Table S1. Isoflavone composition in non-enzymatically treatment SPIEE and aglyconization SPIEE.

	SPIEEE	Aglyconization SPIEE
Isoflavones (mg/100g)*		0.0
Daidzin	1.1	0.0
Glycitin	0.3	0.0
Genistin	2.1	0.0
Malonyl Daidzin	0.8	0.0
Malonyl Glycitin	0.2	0.0
Malonyl Genistin	1.4	0.0
Acetyl Daidzin	0.1	0.0
Acetyl Glycitin	0.0	0.0
Acetyl Genistin	0.1	0.0
Daidzein	0.8	2.5
Glycitein	0.1	0.3
Geistein	0.9	4.1

* Aglycon equivalents at 0.2% concentration solution (in 70% EtOH).

Table S2. Phospholipids in soy lecithin and egg lecithin.

	Soy lecithin*	Yolk lecithin**
Phospholipids (g/100 g)		
PC	31.6	72.5
PE	26.1	14.4
PI	12.7	N.D.***
PS	N.D.	N.D.

*L-α-Phosphatidylcholine from soybean (Sigma-Aldrich, P5638). **L-α-Phosphatidylcholine from egg yolk (Sigma-Aldrich, P5394). ***Not detected.

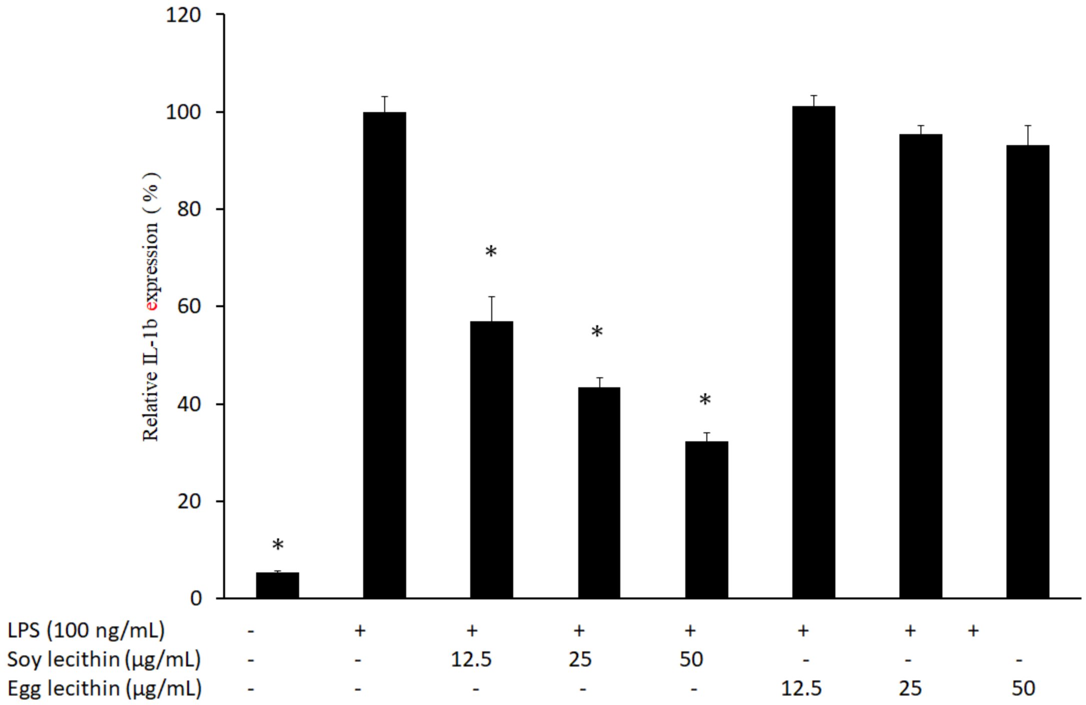

Figure S1. Comparison of the inhibitory effects of soy lecithin and egg lecithin on IL-1β expression in THP-1 cells. Results are shown as values relative to that in the Il-1β treatment group, which was set as 100%. *$p < 0.05$ vs. LPS treatment.

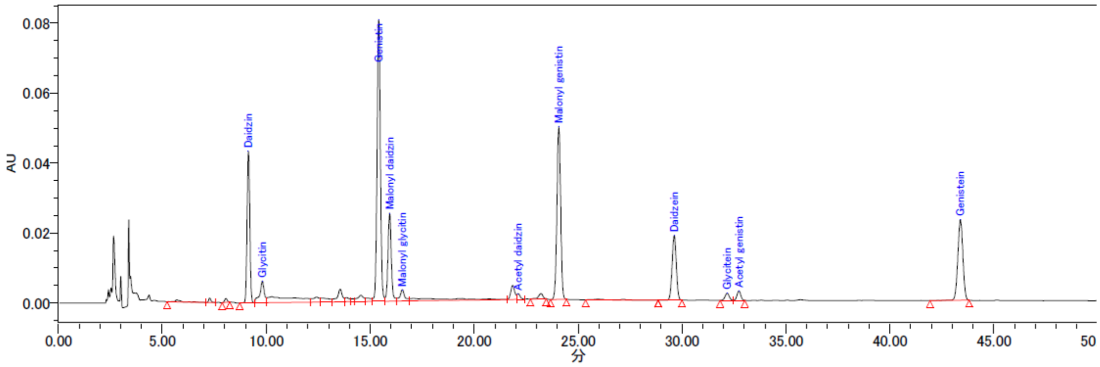

Figure S2. Chromatogram of the separation of isoflavones. Sample: SPIEE. Injection volume: 10μL. Analysis Time: 50min. Analysis Method: 254nm.
